# Exploring academic teachers perspectives regarding the impact of using medical simulation in dentistry pre- and post-COVID-19 pandemic: a qualitative study

**DOI:** 10.1186/s12909-023-04586-6

**Published:** 2023-09-04

**Authors:** Izabela Mamcarz, Katarzyna Sarna-Boś, Renata Chałas, Jarosław Sobieszczański, Wojciech Świątkowski, Luciano Augusto Cano Martins, Kamil Torres

**Affiliations:** 1 Chair of Medical Education, Simulation Laboratory for Patient Safety, Medical University of Lublin, 4 Chodzki Street, Lublin, 20-093 Poland; 2 Department of Dental Prosthetics, Medical University of Lublin, 6 Chodzki Street, Lublin, 20-093 Poland; 3 Department of Oral Medicine, Medical University of Lublin, 6 Chodzki Street, Lublin, 20-093 Poland; 4 Preclinical Dentistry Lab, Medical University of Lublin, 6 Chodzki Street, Lublin, 20-093 Poland; 5 Chair and Department of Dental Surgery, Medical University of Lublin, 6 Chodzki Street, Lublin, 20-093 Poland; 6 Department of Dental and Maxillofacial Radiodiagnostics, Medical University of Lublin, 6 Chodzki Street, Lublin, 20-093 Poland; 7 Chair of Medical Education, Medical University of Lublin, 4 Chodzki Street, Lublin, 20-093 Poland; 8 Department of Plastic and Reconstructive Surgery and Microsurgery, Medical University of Lublin, Lublin, 20-059 Poland

**Keywords:** Medical simulation, Academic teachers’ perspective, Dentistry education, COVID-19 pandemic

## Abstract

**Background:**

Medical simulation allows for the achievement of many educational goals and the continued education of some practical skills. The COVID-19 pandemic’s restrictions have led to a major increase in dental education simulations. The aim of this study was to analyse the perspectives of academic teachers towards dental simulation, their concerns and evaluation of this teaching method, as well as their opinion on the use of medical simulation during the COVID-19 pandemic.

**Method:**

A focus study was conducted in a group of 5 academic teachers, comprising 10% of academic teachers of a Dental Faculty using simulation techniques. Prior to and during the COVID-19 pandemic, the interviewed teachers had expertise with medical simulation in dentistry education methods. A facilitator used pre-planned, open-ended questions about the use of simulation in dentistry also with regard to the COVID-19 pandemic period. The group discussion has been managed, monitored, and recorded. The data analysis model was based on Braun and Clarke’s six phases of thematic analysis. Five thematic domains/fields were evaluated: (1) Simulation as a didactic method; (2) Simulation during COVID-19 pandemic; (3) General observations and expectations with regard to simulation; (4) Teachers in simulation; (5) Concerns in relation to simulation. Two researchers analysed the data.

**Results:**

Based on interviewed teachers’ perspective the simulation allows students to learn basic and complex skills providing the repeatability of the procedures performed. During Covid-19 the simulation methods undoubtedly filled the gap in the training of future dentists. However, interviewed teachers pointed out the high cost of the methods dictated by the need to prepare the simulation environment at a high level, in order to reflect the real clinical situation.

**Conclusions:**

The use of simulation methods requires adequate preparation of academic teachers, continuous education and updating of knowledge in the field of medical simulation. The COVID-19 pandemic significantly influenced the growth of dental education simulation techniques as well as staff knowledge of the usage of medical simulation.

**Supplementary Information:**

The online version contains supplementary material available at 10.1186/s12909-023-04586-6.

## Background

The perspective of academics regarding working with simulation methods seems to be very important considering organizing classes and planning education in dentistry [1, 2]. Medical simulation gives teachers the opportunity to consciously manoeuvre in the educational environment and shape it freely. Teachers can choose what aspects of their subject they want to train and continuously improve their work over time [3]. While supporting students in learning from their mistakes and fostering critical thinking, prior experience with the simulation methodologies and information from dentistry academic teachers are essential for efficiently achieving the educational goals using simulation methods [4–6].

In the Simulation Based Education (SBE) method, teachers and the technical staff in charge of the equipment preparation, service and efficient operation of IT systems in simulation centres is also crucial [3]. Despite the increasing quality of simulation methods over time, there are dental treatment elements that are difficult to reproduce such as the anatomical variability of the patient’s cranium, reflexes, individual behaviour, bleeding, salivation and sweating [7]. Also, the simulation is based on professionally prepared simulation rooms, advanced devices, including wide range of sophisticated digital machines and tools that require significant initial outlays as well as regular service which can increase the cost while implementing this environment [7]. However, it’s crucial to recognize that simulation is a rapidly evolving area of education, and that such restrictions might be scaled back or even eliminated.

Due to the COVID-19 restrictions, both personal and social communication have been limited to virtual interactions or shifted to altered clinical spaces [8]. The medical simulation in both on-site and online classes has also emerged as the true clinical teaching substitute [9–13]. A perspective from academic teachers on the importance of medical simulation and its effect on dental education may indicate the actual level of use of simulation especially in a challenging situation [14]. This study aimed to analyse the opinion of academic teachers towards dental simulation, their concerns and evaluation of this teaching method. Teachers opinion on the use of medical simulation during the COVID-19 pandemic was also analysed.

## Methods

Ethical approval for the survey was granted by the Local Bioethics Committee (Approval no. KE-0254/155/2021).

A focus study was organized as it is known as a powerful research tool thanks to the use of qualitative methods to learn descriptive information from the interviewed teachers, which can be very helpful in learning about the researched phenomenon [15, 16]. Our research paradigm was constructivism, as we were aiming to learn about the opinion and perspectives of research group in relation to the specific phenomenon of using medical simulation in teaching.

When this study was carried out, approx. 50 academic teachers at the participating Medical University were using simulation techniques (for example Phantom Head Simulators, Virtual Reality (VR) Dental Simulators, Dental Simulated Patients, Dental Surgical Simulation, Dental Simulation Software). To cover the requirements for focus groups, a representative sample of 10% of the scholars (3 males, 2 females) with a minimum of 15 years of experience working as academic teachers were surveyed [17]. Their work experience with medical simulation methods was at least 5 years. The participants were a group of specialists in the field of utilizing simulation methods in dental education. The study process was rich in mutual interactions, exchange of experiences, and discussions, which contributed to obtaining reliable research material. The group discussion was moderated, monitored, and recorded by a facilitator (IM), according to pre-planned open-ended questions (Attachment 1) on the use of simulation in dentistry and taking into account the teaching of dentistry during the COVID-19 pandemic. The meeting was organized into the following steps: setting scenes and grounded rules, individual introduction, opening topic discussion, proper discussion and ending the discussion. The group discussion duration was approximately 90 min. The interaction between participants was important in generating the data which could encourage deeper discussion [18].

**Fig. 1:**
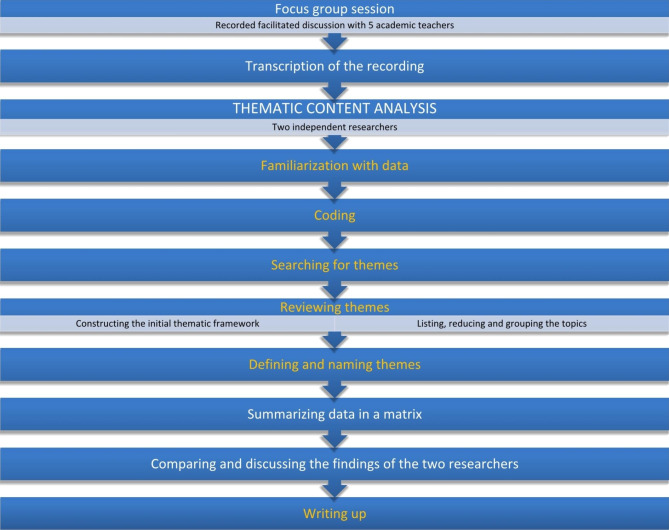
An illustration of Braun and Clarke’s thematic analysis approach in the data collection and analysis model (adapted from Braun and Clarke’s, 2013)

As part of the described qualitative study, a thematic analysis was conducted. The qualitative analysis was based on a Braun and Clarkes’s six phases of thematic analysis as the data analysis strategy [19, 20]. According to the authors’ approach, the method of conducting thematic analysis should not be treated as a linear model. They present a reflective thematic analysis approach, emphasizing the researcher’s reflexivity and subjectivity. Therefore, the phases described by Braun and Clarke have been incorporated into the data analysis model used in this article. The model is presented in Fig. 1.

During the conducted focus group study, the session was recorded and then the recording was transcribed. The text was subjected to thematic analysis by two independent researchers. At this stage, the text from the transcription was initially read to gain familiarity with the addressed topic, and then it was carefully reviewed. During this process, the researchers conducted a thematic analysis, seeking and identifying issues, topics and concepts in the text that were relevant to describing the phenomenon under study. The identified issues were later grouped into sets of topics, resulting in main themes. Thematic analysis identified five themes from the analyzed content: (1) Simulation as a didactic method; (2) Simulation during COVID-19 pandemic; (3) General observations and expectations with regard to simulation; (4) Teachers in simulation; (5) Concerns in relation to simulation. Within the first theme “Simulation as a didactic method”, two sub-themes were covered: advantages and disadvantages of the simulation methods. The transcription text was reanalyzed with this proposed categorization in mind. These themes helped organize the discussed content and attempted to present general conclusions from the study. The basis for grouping the analyzed topics into five main thematic categories was the way in which the participants presented their perspectives. The thematic analysis of the text indicated the possibility of such grouping of the discussed issues. Within each thematic group, the obtained research content was further analyzed in terms of the addressed topics and presented in the study. The conducted study allowed for the achievement of the main research objective, which was to illustrate the subject from various viewpoints and attempt to outline a shared understanding related to the use of simulation in dental education.

## Results

The examples of transcript answers for each studied theme are presented on Table 1. The results are also presented according to the overall perception of the teachers answers throughout the themes.

**Table 1:** Example of the statements for each theme

Themes:	Examples of the statements:
Simulation as a didactic method: A. Advantages B. Disadvantages	• *“Possibility of making mistakes. Without consequences”* • *“It allows us to teach basic skills. (…) Simple manual activities. “* • *“(…) So this communication (…) the first stage in treatment can also be perfectly trained here.“* • *“(…)Here, on the other hand, the whole infrastructure, technology, materials must be prepared on an ongoing basis. And they are consumed frequently as well.“* • *“(…) we are not able to replace everything with simulation,“*
Simulation during COVID-19 pandemic	• *“I think this role is very important because there was a time when the treatment of patients by students was impossible and the simulation would fill that gap. So it’s the kind of thing that even in an emergency patient shortage situation can provide continuity of education (…).“* • *“Certainly this pandemic has changed the perception that when something happens (…)* • *we have an alternative.”* • *“(…)This pandemic shows that this safety is important, and it can be taught in the simulation (…).“*
General observations and expectations with regard to simulation	• *“(…) And we, therefore, are only a step behind because certain changes have occurred globally on dentistry. But we can’t say it’s not there because it wasn’t. There has been some preparation in parallel with medicine faculty.“* • *“So it’s a matter of smart management and collaboration.“* • *“(…) some things while studying dentistry we didn’t practice at all. We actually learned later when we went to work.“*
Teachers in simulation	• *“(…) there were groups of teachers where all this was denied, it was unnecessary (…).“* • *“Too much electronics, as some people say.“* • *“We still need to educate the staff so that they understand the meaning of the simulation* • *and know how to implement it in a credible way.”*
Concerns in relation to simulation	• *“(…)What we have already said, so that this simulation does not develop like this, that one day it replaces the clinic.“* • *“Verification of these activities, the ones we set up for ourselves what they are supposed to learn, and then verification that they have actually acquired these activities.“* • *“I’m just afraid to have enough money for everything, because it costs a lot (…).”*

### Theme (1): Simulation as a didactic method

Dental simulation as a didactic tool has both benefits and drawbacks, according to the interview data.

### Theme (1a): Advantages

Simulated teaching has the undeniable advantage of providing the chance to make mistakes without having them negatively impact the patient. Additionally significant factors were addressed, including risk minimization and epidemiological safety.

Interviewed teachers noted that by conducting a certain exercise repeatedly, simulation enables the tasks to be implemented as training. The simulation allows students to learn basic skills and simple manual activities by providing the repeatability of the procedures performed. Furthermore, in their point of view, simulation techniques allow for the teaching of more complex skills and aid in the development of the capacity to carry out more sophisticated activities in the last years of the study and after the study.

The possibility of shaping appropriate attitudes and teaching the principles of savoir-vivre was another aspect that interviewed teachers paid attention to. Proper dentist-patient, dentist-another dentist, dentist- dental assistant communication is a very important skill, which can also be shaped and developed thanks to the student’s participation in medical simulation classes. In their responses, the interviewed teachers focused on how well students could communicate with patients, discuss unpleasant news, or deal with patients who are seen to be challenging. The interviewed teachers highlighted that it is possible to use the aspect of interdisciplinary work as a part of the dental course using medical simulation. Additionally, they emphasized the ability to create teams of students from different medical faculties, who collaborate to realize one simulation scenario. Academic classes using simulation also provide an opportunity to learn teamwork as well as to safely practice skills as part of a therapeutic team under controlled and repeatable conditions. Example answers are transcribed below:

*“Another thing is still collaboration with other departments. Not only within our dentistry, but with the medical faculty, with the nursing faculty, with the pharmacy department (…).“*

*“(…) a student could learn even cultural phrases to a person who is subordinate to him in the course of work (…).“*

The interviewed teachers contend that using medical simulation as a teaching strategy is effective, rational, and better equips students to visit the clinic and interact with real patients. There were additional suggestions made during the interviews that teaching with the use of simulation techniques could be beneficial in confirming the students’ competency and skill.

### Theme (1b): Disadvantages

The main disadvantage pointed out by the teachers is the high cost of education using simulation. Specialized equipment, consumable materials can be expensive and should be carefully prepared for each student. This is dictated by the need to prepare the simulation environment at a high level, to reflect the clinical conditions as closely as possible.

It was also pointed out that aspects such as the vomiting reflex or bloody procedures would be difficult to plan for in a simulation exercise, which at the same time may constitute a limitation in the planning and organization of simulation classes.

Among the statements of the interviewed teachers, there were statements that it is impossible to replace all dental training with simulation classes. The role of patient contact was particularly emphasized. In addition, some content, aspects taught in the course of dental education require the inclusion of other teaching methods. At the same time, it does not exclude the great role of medical simulation in teaching dentistry and the possibility of complementary use of simulation along with other teaching methods.

A particularly important theme emphasized by the academics was the attitude of teachers teaching dentistry using simulation techniques. They also emphasized the need for faculty to be properly educated as seen in the following example answers:

*“We still need to educate the staff so that they understand the meaning of simulation and know how to implement it in a reliable way.*

*“But it seems to me that there is a need for such five-stage training, specific scenarios, or in a particular field, refined.“*

### Theme (2): Simulation during COVID-19 pandemic

The COVID-19 pandemic forced the implementation of methods designed to sustain the educational process despite the prevailing restrictions. Interviewed teachers highlighted the significant role of simulation teaching during the COVID-19 pandemic. They described it as indispensable, irreplaceable and claimed that simulation undoubtedly filled the gap in the training of future medics.

Interviewed teachers noted that university teachers’ perceptions of medical simulation may have changed during the COVID-19 pandemic. The situation related to the COVID-19 pandemic played an important role in shaping an attitude of university teachers towards job security. They also found that medical simulation could provide a solution and be helpful in difficult situations such as that associated with the COVID-19 pandemic.

Moreover, the teachers pointed out that it is important to improve teaching using simulations in order to be prepared for such a situation as the COVID-19 pandemic has become. With proper preparation, implementation and development of medical simulation methods applied in academic units, it will be possible to ensure high quality of education as seen:

*“(…)And simulation at this point was a natural solution, a kind of safety, a valve that gave the possibility that something might happen. (…) So this simulation at this point seems to be the only thing we have. And the only thing left for us is to improve it (…).*

### Theme (3): General observations and expectations with regard to simulation

During the interview, the participants pointed out that simulation in teaching dentistry has been used before, as simulation includes teaching dentistry on phantom heads. However, they have not always been intensively developed and improved. Simulation workstations (dental unit and head and trunk) allow students to perform clinical procedures on manikins under conditions that replicate those in a doctor’s office.

The interviewed teachers highlighted that there is a need for rapid development of medical simulation implemented in dental studies, especially in relation to the level it has reached in medical studies, where it has already been implemented. They also observed that medical simulation provides an opportunity to practice practical skills more often than in the past when it was not as popular.

The interviewed teachers pointed out that a good solution could be combining computer simulation, virtual simulation and standardized patient. They also mentioned the need for the development of computer-based solutions in dentistry, while noting that simulation cannot replace clinical treatment at every stage.

It was further noted that the issue of simulation funding in dentistry, which was discussed as a drawback of simulation, is a matter of good management and governance:

*“So, it’s a matter of smart management and collaboration.“*

### Theme (4): Teachers in simulation

The analysis of the data collected during the focus study shows that according to some of the interviewed teachers, teachers who have not instructed dental students using simulation techniques (such as Phantom Head Simulators, Virtual Reality (VR) Dental Simulators, Dental Simulated Patients, Dental Surgical Simulation, Dental Simulation Software) often find it tedious or unnecessary. It is sometimes negatively evaluated by lecturers and not treated as a reliable teaching method. As examples of reasons for such an attitude of academic teachers to simulation, those surveyed indicated, among other things, the need to learn new techniques, the need to change the view of the teaching process and change their current habits.

The high level of technological advancement of simulation, which is faced by instructors in practice, also seems to be of significance. Therefore, it is important to know the teachers’ attitude towards simulation, their possible prejudices or doubts related to the use of this method in teaching dentistry. This may translate into the effectiveness of using medical simulation in teaching dentistry, as well as the level of satisfaction from working with medical simulation.

At the same time, the interviewed teachers point out that teachers’ attitudes towards the use of simulations in teaching dentistry may develop into a more positive one over time. Indeed, it appears that according to their experiences and observations, initial reluctance may later change to a positive attitude as seen in the quotes below:

*“They were simply individuals who did not want to cooperate.”*

*“Too many electronics, as some people say.“*

*“(…) However, when it started after a year, it suddenly turned out that there was simply not enough time and space, because everyone wanted to have classes here in the simulation.“*

Interviewed teachers further emphasized the importance of the soft skills of the educators teaching dentistry using simulation techniques. Among the soft skills listed above are: communication skills, empathy and patient-centered approach, critical thinking and problem-solving, collaboration and teamwork, professionalism and ethical conduct. This seems to be important because they often interact with students, perform important processes for medical simulation classes such as prebriefing and debriefing, provide feedback, as well as operate low or high-fidelity simulators themselves.

In relation to this, the need to have such skills and competences as patience, communication skills, organizational skills, or the ability to operate the equipment used during the classes appeared in the opinions of people surveyed. The interviewed teachers emphasized that the teachers using medical simulation should be patient, communicative, have organizational skills, demonstrate knowledge of technology and knowledge of simulation as a method of imitating reality.

According to the interviewed teachers, simulation requires a lot of creativity and innovation on the part of the teacher to make full use of its possibilities. Therefore, there is a constant need for training for instructors. Simulation requires a constant development from educators.

### Theme (5): Concerns in relation to simulation

During the conducted focus study interviewed teachers also referred some concerns about the use of medical simulation in teaching of dentistry. These were related to the need of maintaining appropriate conditions, or the implementation of assumptions that will make medical simulation a helpful and effective method of teaching. The academic teachers participating in the study highlighted, among other things, such aspects as adequate preparation for teaching in simulated conditions, or the management of simulation as a teaching method.

One of the concerns expressed by interviewed teachers during the focus study was that medical simulation would replace clinical classes with real patients. It was also pointed out that a so-called cut-off point from simulation, e.g., verification of students’ skills before entering the clinic, is needed and important.

*“(…)What we have already said, so that this simulation does not develop like this, that one day it replaces the clinic.“*

In their opinion, this could be important for increasing the quality of teaching practical activities and achieving a higher level of student preparation. In addition, concern was also expressed about the financial possibilities of implementing didactic activities using medical simulation in dental studies.

## Discussion

The current focus study aided in defining the key benefits and drawbacks of medical simulation, as well as in learning about the experiences, hopes, and worries raised during the study in relation to the use of medical simulation in practice. It also helped to identify any potential changes that might be required in the way dental teaching is carried out using medical simulation. In addition, the role of teachers conducting didactic classes using medical simulation has also been defined, as well as the situational context related to the COVID-19 pandemic has been characterized.

In line with the previous studies [21, 22], interviewed teachers emphasized that the techniques from medical simulation have been used for a long time, However, as simulation technology advances, it becomes conceivable to broaden its application in teaching dentistry. The teachers who were interviewed expressed that the use of medical simulation in dentistry education has led to notable advancements in terms of the accessibility and effectiveness of teaching methods.

According to the teachers who were interviewed, one of the primary benefits of using medical simulation in dentistry is the ability to make errors without facing any real-world repercussions, while still having the opportunity for introspection and learning from those mistakes. In simpler terms, these educators believe that the key advantage of dental medical simulation is the chance to practice and learn from errors in a safe environment. Also, other studies show the importance of this aspect in the use of medical simulation in teaching [23, 24]. The opportunity of task-based training with repeatability was also highlighted in order to teach basic skills in preclinical conditions. It was noted that medical simulation in dentistry does not have to be limited only to preclinical training of students, but also be an attractive teaching method in the process of postgraduate education, as also indicated by other research results [25, 26].

In addition to the increasing possibilities brought by the developing technology used in simulation, it was indicated that it is advisable to introduce communication skills between a doctor and a patient into teaching dentistry, but also in a dental team, e.g. with a dental hygienist, which has also been report previously in other studies and systematic reviews [27–29]. The environment of the simulation centre for this type of task are optimal. In addition, it was noted that conducting classes together with students of other faculties creates the opportunity to teach in the context of the ability to conduct consultations, express opinions and treat patients requiring multi-specialist consultations. Summing up the role of medical simulation in teaching dentistry, the possibility of its use in teaching dentistry was clearly appreciated.

The costs associated with providing access to disposable parts, and the requirements for constant servicing and supervision were emphasized as disadvantages of simulation in dentistry. It was also found that the environment of the medical simulation in dentistry cannot always be sufficiently realistic to imitate clinical conditions, especially simulating the work of a dentist in oral cavity. Lin et al. (2017) emphasized that economic evaluation in simulation based medical education can be helpful to determine the optimal use of resources [30].

Members of the studied group clearly emphasized the important role of simulation in dentistry as a didactic method improving the effects in the education of dentists. Substantial change in the approach of academic teachers to medical simulation in dentistry and a gradual increase in acceptance of its usage as a didactic tool were noticed. Additionally, it was said that, like any didactic method, it needs ongoing development of both the techniques and tools used as well as the instruction of the instructors in charge of this area’s didactics. In order to adequately employ this didactic style, teachers must be committed, patient, and continually trained. It also seems crucial that those in charge of the didactic process are aware of the many advantages and restrictions connected to the use of medical simulation in the classroom.

Medical universities have become a place of transformation in the teaching methods of dental students during COVID-19 pandemic [31, 32]. According to some teachers, the decision to implement remote classes in dentistry, a field known for its emphasis on practical skills, was seen as a measure of last resort. In other words, these educators believed that resorting to remote learning in dentistry, despite its practical nature, was a necessary step taken only when no other options were available.

This had a positive impact on the safety of students and teachers, especially in the initial phases of the pandemic – before the widespread use of vaccinations, but significantly limited the possibility of practical performance of therapeutic procedures and contact with the patient [33, 34]. The dynamically changing epidemiological situation in the country and worldwide forced the introduction of new didactic methods, in some way supplementing the deficiencies associated with limited access to clinical classes [35, 36]. Medical simulation has enabled students to participate in practical classes and manually master dental procedures in simulated conditions, preparing in a better way for clinical classes in later semesters of study. The scope of performed procedures and individual subjects carried out in medical simulation conditions in the period 2019–2021 has been significantly expanded, including procedures previously performed only in clinical settings [33, 37].

The introduced changes in dental education and involvement of medical simulation in the didactic process require both adaptation of the students and teaching staff. Changes in the dental training, which were initiated by the COVID-19 pandemic, are related to the didactic methods used today, the equipment used or technical conditions that are provided as part of classes using medical simulation. It seems important to develop simulation in dentistry, to determine its safe scope throughout the didactic process, so that it does not become just a “supplement”, but a modern method to improve the effectiveness and attractiveness of dentistry studying.

The results of the focus study clearly indicate increasing rank of medical simulation in dentistry in recent years. The period of the COVID-19 pandemic forces the increasing appliance of medical simulation, also in dentistry, while ensuring safe conditions for the training of simple manual skills, as well as the implementation of scenarios required for a specific curriculum [38]. The study clearly concluded that medical simulation cannot completely replace clinical practice with a patient.

The conclusions reached about medical simulation in dental teaching appear to coincide with the results published by other authors [39]. Striving to reduce the risk of transmission of COVID-19 virus infection in subsequent waves of the pandemic forced a significant increase in the use of medical simulation, refining and improving its technical aspects in dentistry. In addition, hybrid teaching methods combining stationary teaching with e-learning methods have gained acceptance among teachers.

As a limitation of a study, broadening of the research and sharing of the student feedback related to simulation in dentistry, as well as expanding the study group to multiple academic centres should be considered in the future research. It would be valuable to compare the opinions and perspectives of academics working with simulation methods in teaching dentistry at other universities, in other countries, or to compare different groups of interviewed teachers, for example, students, teaching managers, practicing dentists. Additionally, it would be interesting to compare opinions on teaching using simulation methods and motivation to teach using these methods with other variables like the general motivation of teaching staff to work, undertake development, additional courses. Analysis of such data would help to develop a new or modified dental curriculum based on hybrid learning combining e-learning, medical simulation and clinical teaching methods in a way that reduces the negative impact of the pandemic [40, 41].

## Conclusion

The use of simulation methods requires adequate preparation of academic teachers, continuous education and updating of knowledge in the field of medical simulation. The COVID-19 pandemic significantly influenced the growth of dental education simulation techniques and expanded staff knowledge of medical simulation usage.

### Electronic supplementary material

Below is the link to the electronic supplementary material.

Supplementary Material 1

## Data Availability

The datasets used and/or analysed during the current study are available from the corresponding author on reasonable request.
