# Supplementary material for: Exploring academic teachers perspectives regarding the impact of using medical simulation in dentistry pre- and post-COVID-19 pandemic: a qualitative study
Source: BMC Med Educ. 2023 Sep 4;23:633. doi: 10.1186/s12909-023-04586-6 (PMC10478263; doi:10.1186/s12909-023-04586-6)
Supplement: Supplementary file 1 — Supplementary Material 1 [file 12909_2023_4586_MOESM1_ESM.docx]

**Title:** Exploring Academic Teachers Perspectives regarding the Impact of Using Medical Simulation in Dentistry Pre and Post-COVID – 19 Pandemic: A Qualitative Study

**Keywords:** medical simulation, academic teachers’ perspective, dentistry education, COVID-19 pandemic

**Running Title:** Teachers' perspectives on using medical simulation

**Authors:**

Izabela Mamcarz, Katarzyna Sarna-Boś, Renata Chałas, Jarosław Sobieszczański, Wojciech Świątkowski, Luciano A. C. Martins, Kamil Torres

Izabela Mamcarz, corresponding author

Department of Didactics and Medical Simulation, Medical University of Lublin

4 Chodzki Street, 20-093 Lublin, POLAND

[izabelamamcarz@umlub.pl](mailto:izabelamamcarz@umlub.pl) , +48 81 448 59 30

Katarzyna Sarna-Boś

Department of Dental Prosthetics, Medical University of Lublin

6 Chodzki Street, 20-093 Lublin, POLAND

[katarzynasarnabos@umlub.pl](mailto:katarzynasarnabos@umlub.pl), +48 81 502 17 80

Renata Chałas

Department of Oral Medicine, Medical University of Lublin

6 Chodzki Street, 20-093 Lublin, POLAND

[renata.chalas@umlub.pl](mailto:renata.chalas@umlub.pl)

Jarosław Sobieszczański

Preclinical Dentistry Lab, Medical University of Lublin

6 Chodzki Street, 20-093 Lublin, POLAND

[jaroslawsobieszczanski@umlub.pl](mailto:jaroslawsobieszczanski@umlub.pl)

Wojciech Świątkowski

Chair and Department of Dental Surgery, Medical University of Lublin

6 Chodzki Street, 20-093 Lublin, POLAND

[wojciech.swiatkowski@umlub.pl](mailto:wojciech.swiatkowski@umlub.pl), +48 81 502 17 40

Luciano Augusto Cano Martins

Department of Dental and Maxillofacial Radiodiagnostics, Medical University of Lublin

6 Chodzki Street, 20-093 Lublin, POLAND

[luciano.cano-martins@umlub.pl](mailto:luciano.cano-martins@umlub.pl), +48 81 502 18 03

Kamil Torres

Department of Didactics and Medical Simulation, Medical University of Lublin

4 Chodzki Street, 20-093 Lublin, POLAND

[kamil.torres@umlub.pl](mailto:kamil.torres@umlub.pl), +48 81 448 59 30
